# Role of Reference Frames for a Safe Human–Robot Interaction

**DOI:** 10.3390/s23125762

**Published:** 2023-06-20

**Authors:** Alberto Borboni, Roberto Pagani, Samuele Sandrini, Giuseppe Carbone, Nicola Pellegrini

**Affiliations:** 1 Mechanical and Industrial Engineering Department, Università degli Studi di Brescia, Via Branze 38, 25123 Brescia, Italy; roberto.pagani@unibs.it (R.P.); nicola.pelegrini@unibs.it (N.P.); 2 STIIMA-CNR-Institute of Intelligent Industrial Technologies and System, National Researcher Council of Italy, 00185 Roma, Italy; samuele.sandrini@stiima.cnr.it; 3 Department of Mechanical, Energy and Management Engineering, Università della Calabria, Via P. Bucci, Edificio Cubo 46C, Arcavata di Rende, 87036 Rende, Italy; giuseppe.carbone@unical.it

**Keywords:** safety, reference frame mapping, human–robot interaction

## Abstract

Safety plays a key role in human–robot interactions in collaborative robot (cobot) applications. This paper provides a general procedure to guarantee safe workstations allowing human operations, robot contributions, the dynamical environment, and time-variant objects in a set of collaborative robotic tasks. The proposed methodology focuses on the contribution and the mapping of reference frames. Multiple reference frame representation agents are defined at the same time by considering egocentric, allocentric, and route-centric perspectives. The agents are processed to provide a minimal and effective assessment of the ongoing human–robot interactions. The proposed formulation is based on the generalization and proper synthesis of multiple cooperating reference frame agents at the same time. Accordingly, it is possible to achieve a real-time assessment of the safety-related implications through the implementation and fast calculation of proper safety-related quantitative indices. This allows us to define and promptly regulate the controlling parameters of the involved cobot without velocity limitations that are recognized as the main disadvantage. A set of experiments has been realized and investigated to demonstrate the feasibility and effectiveness of the research by using a seven-DOF anthropomorphic arm in combination with a psychometric test. The acquired results agree with the current literature in terms of the kinematic, position, and velocity aspects; use measurement methods based on tests provided to the operator; and introduce novel features of work cell arranging, including the use of virtual instrumentation. Finally, the associated analytical–topological treatments have enabled the development of a safe and comfortable measure to the human–robot relation with satisfactory experimental results compared to previous research. Nevertheless, the robot posture, human perception, and learning technologies would have to apply research from multidisciplinary fields such as psychology, gesture, communication, and social sciences in order to be prepared for positioning in real-world applications that offer new challenges for cobot applications.

## 1. Introduction

Robots are a pivotal element for modern industry due to their reliability, flexibility, and collaborative usage applications [1]. Collaborative robots, cobots, as defined in ISO/TS 15066:2016, are the cutting edge in industrial robotics, based on the shared abilities between workers–robots and what is foreseen by the Industry 4.0 paradigm. Human–robot collaboration is required to combine the accuracy and reproducibility of robots with the flexibility and adaptability of workers in a mutual task accomplishment. Moreover, collaborative robots are designed to actively interact with humans. According to [2], there are five levels of possible interactions: cell (not genuine cooperation, because the robot is in a cage); coexistence (workspace not shared); synchronized (only one present at a time); cooperation (shared workspace, non-simultaneous tasks, and separate object); and collaboration (simultaneous work on the same product). To ensure the workers’ safety, they are equipped with several sensors and force-limiters that replace the safety fences of standard industrial robotic work cells. Collaborative programming methods are predominantly agile and may be adapted to the dynamical environment accordingly [3]. Guaranteeing safe interactions, robots should be intuitively operated by humans. Safety is mandatory in the design of the systems for workplaces where humans work in conjunction with robots. An agreement on standards is a prerequisite to prove the safety aspect [4], as regulated by the certification process of the Machinery Directive. Three categories are classified: (i) The type A standard is expressing the terminology and methodology elements according to ISO 12100, IEC 61508; (ii) the type B standard defines the safety aspects according to ISO 13849-1, IEC 62061, section B1 and the safeguarding of humans according to ISO 13850, ISO 13851, section B2; and (iii) the type C standard specifies individual safety standards according to ISO 10218-1, which contains the requirements for robot manufacturers, and ISO 10218-2, which is proposed for integrators [5]. The ISO 10218-1 standard expresses collaborative operation requirements, considering that the maximum static force is limited to 150 N and the dynamic power at 80 W at the end effector flange. Consequently, four collaborative models are defined as follows: safety-monitored stop, speed separation monitoring, hand-guiding, and power force-limiting. In the safety-monitored stop, the operator shares the workplace with the robot to perform the manual tasks. The robot can work in the workplace in operator-free time slots [6], and its task remains in active stop mode. When the operator leaves the shared workspace, the robot resumes its execution. In [7], the authors described, in some detail, the current standards applicable to collaborative robots and mobile robots based on a cross-domain validation approach tested with an online toolkit. In the speed separation monitoring model, humans may work alongside robots by ensuring safety by means of several sensors installed on the robot or in the environment. The robot executes its task at maximum speed if the operator is in the green zone, at decreased velocity when in the yellow area, and it stops when the worker accesses the red area. The zone definition is based on a collision risk analysis and the human–robot mutual distance. These volumes are often monitored by scanners and/or vision systems. In reference [8], safety was achieved by recognizing the user’s facial expressions by means of deep learning. In [5], a plethora of solutions for collision-free robot motions from the surrounding objects were presented and discussed. The application in [9] was a workstation-safe operation without fences, where the limit was provided by infrared sensors used to monitor the operators’ locations. The work in [10] introduced a real-time speed separation monitoring system based on the human–robot direct distance measure. The adoption of multi-sensing architecture to perceive obstacles within robot operative zones and respond to the planner with Kineo-SW for a fast trajectory selection was studied in [11]. In [12], the authors discussed a collaborative application based on a dynamic system that varied the industrial robot velocity by using redundancy from Kinect and a certified system that was activated if the malfunction of the primary detector happened. The authors of [13] presented a controller for safe robot–worker coexistence. Safe control approaches for definite sensing equipment, such as observation systems or depth cameras, have been described in relevant works. Hand-guiding models characterize the scenario in which the subject can teach the autonomous system a motion by moving the end effector without an ad hoc GUI. This complex collaborative configuration requires machines equipped with both safety-monitored stop and speed-separated functionalities. The power force-limiting approach requires devoted equipment and controls to manage the collision with humans. The limitations are related to the motor power and force exerted by the robot. A review of human–robot interactions was presented in [14] describing injuries, their classification, and a report of the collision types. A mechanical force element that protects workers from impacts was presented in [15]. The work in [16] described a control strategy combined with an adaptive damping system. Furthermore, an experience-based approach was proposed in [17]. The method exploited neural network architecture trained on a dataset of sensory data to estimate the robot’s force. Residual models and multi-sensors were studied in [18] for the real-time assessment of the exerted force and impact area detection.

Although the literature shows promising methods and significant results in addressing safe HR interaction strategies to achieve effective and collaborative applications, further studies are required to evaluate novel techniques for reference frame modeling. In particular, the state-of-the-art technology shows that several safe HR methods are based on high dynamics, minimizing the human–robot distance needed to carry out robot decisions with unknown moving entities (obstacles and humans) [19,20]. In this way, the development of a human–target observability model focused on the reference frame may represent a novel paradigm of analytical distributed control. A human being accepts three types of spatial reference frames to describe unstructured movements: egocentric, allocentric, and route-centric [21]. The axes of the egocentric reference frame are along the vertical, longitudinal, and lateral directions. The axes of the allocentric reference frame depend on the perception of the entity associated with this reference. In the route-centric reference frame, an axis corresponds to the route. During the motion gestures, the human moves from one reference frame to another according to a real-time collision-free analysis. Consequently, the temporal definition of the adopted reference frame is highly discontinuous and nonlinear. The human perception of the environment and the influence of existing entities in an unstructured space need to be considered. Humans identify themselves as the observed agent; thus, a reference frame is placed on them to predict their movements. The main design specifications for reference frame selection approaches are summarized according to the following criteria and shown in Table 1.

The first column RF is the type of reference frame, the second column is the investigated application, the third column MP designates the main purpose of the research, the fourth column AA indicates the adaptation ability, the fifth column D specifies the type of disturbance considered, the sixth column KC lists the key characteristics of the research, and finally, the last column Ref enumerates the categorized references. In the investigated scenarios, different sensor feedbacks are presented ranging from medical to surgical and autonomous mobile robots. They are needed for 3D mapping; hence, the signals’ management is a key aspect to consider. The usage of an adequate signal loop is the first requirement; moreover, the interface communication between humans and robots needs to be accurately defined, ensuring coherent interpretation to avoid any harmful impacts. Finally, the disturbance features and their representation are directly related to the complexity of the model and the risk of task accomplishment. All previous aspects support the online reference transformation and reference transfer strategies in a global environment for collaborative applications.

This paper aims at proposing a control approach for proper reference frame identification and representation that can guarantee safety with a dynamic formulation of the workspace accessibility. The main contribution is a proposal of a novel quantitative measure of safety based on the robot configuration and its dynamic performance.

The paper content is organized as follows: Section 2 addresses the problem formulation of human–robot interactions. Section 3 describes the proposed procedure, with a focus on the materials and methods. In Section 4, a case study of a human–robot collaboration is proposed by referring to an experimental setup and tests with a seven-DOF anthropomorphic arm; in addition, the psychometric results obtained through a questionnaire from 40 volunteers are presented. Section 5 reports some discussion on the obtained results, and finally, the Section 6 summarizes the findings, limitations, and possible future works as the outcomes of this paper.

## 2. Problem Definition

**Figure 1:**
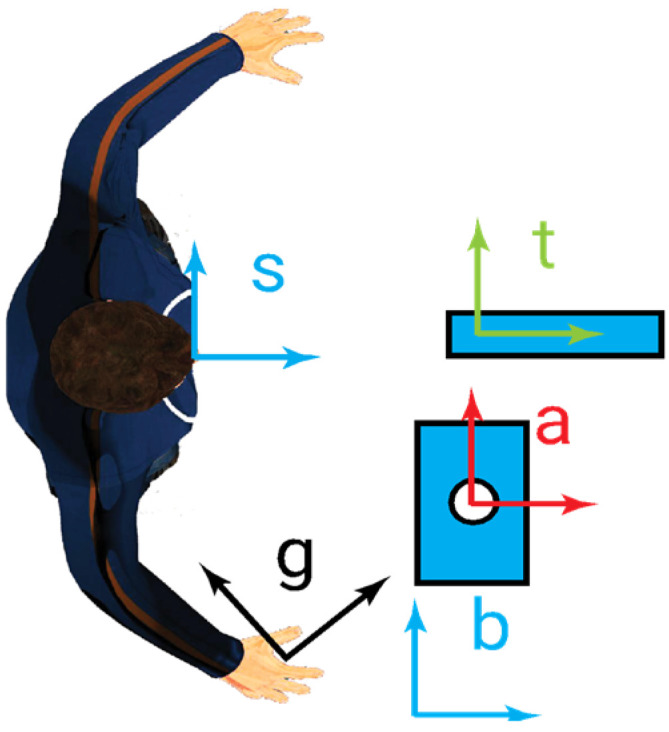
Reference frames ***s***, ***g***, ***t***, ***a***, and ***b*** are, respectively, the sensor, gripper, task, anti-task, and base reference frames.

The proposed problem includes multiple interconnected aspects that are discussed in Section 4. The problem definition considers a human subject working in a fixed position on one or more objects to modify their shapes or assemble them (Figure 1). The human subject works in a working area that is fixed with respect to a fixed reference frame. One or more input areas are present to feed the working activity. One or more output areas are defined to allocate the final result of the working activity. The human subject takes the input objects, usually with the principal hand, then uses the secondary hand to block one object with respect to the fixed reference frame, takes an eventual second object for an assembly activity or a tool for a working activity, then, with the principal hand, executes the procedure; finally, always with the principal hand, the worker puts the final result in the output area. When the action is repetitive, the secondary hand can be used to transfer objects from one area to another area. To improve the accuracy and precision, or to improve the load ability, the subject can adopt instruments to fix, move, assembly, or work the piece. In general, all these actions can be split into elementary actions consisting of aligning a reference frame on the hand to a reference frame on the piece. For the sake of simplicity, we consider only the principal hand (right hand) that acts as a robot gripper or tool, while the secondary hand (left) is used only as help for the principal hand’s actions. Thus, a reference frame ***g*** is set on the hand. The piece is considered as a target; thus, a reference frame ***t*** is set on the piece. The subject observes all the process with his/her eyes. To avoid problems focused on vision and prospective, a sensitive reference frame ***s*** is set between the eyes. If contact with an object must be avoided, a reference frame ***a*** is set on it, where a stands for anti-task. Finally, a fixed reference ***b*** (base) can be adopted to describe the whole system (Figure 1).

**Figure 2:**
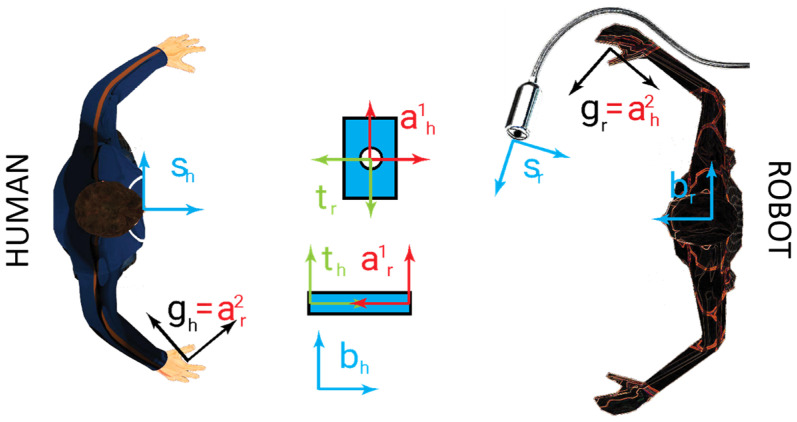
Reference frames in a collaborative layout between a human agent and a robot agent: ***s***, ***g***, ***t***, ***a***, and ***b*** are, respectively, the sensor, gripper, task, anti-task, and base reference frames; the ***h*** and ***r*** subscripts are associated, respectively, with human and robot agents.

When the functional action of the subject is realized in collaboration with another agent, the other agent is endowed with analogous reference systems. We considered a collaborative action between a human agent and a robotic agent; thus, to distinguish the two sets of reference frames, the adoption of the subscript ***h*** is associated with the human reference frames ***g_h_***, ***t_h_***, ***s_h_***, ***a_h_***, and ***b_h_***, and the adoption of the subscript r is associated with the robot reference frames ***g_r_***, ***t_r_***, ***s_r_***, ***a_r_***, and ***b_r_***. For generalization purposes, if multiple reference frames of the same type are adopted for a single agent, a superscript integer number (starting from one) is associated with a frame, i.e., a robotic agent with two grippers can be endowed with references ***g*^1^*_r_*** and ***g*^2^*_r_***. Every single gripper cannot have a single target at the same time. Eventually, different targets can be associated with different actions to be realized sequentially. A single gripper can be requested to avoid different objects during the realization of its action; thus, different anti-target frames can be associated with each gripper, and thus, when multiple grippers are present for a single agent, two superscripts are adopted. The first superscript is associated with the gripper and its target, whereas the second superscript is used only for the anti-target, i.e., ***a*^1,2^*_r_*** and ***a*^2,1^*_r_*** are, respectively, the second anti-target reference frame for the first gripper and the first anti-target reference frame for the second gripper. Different frames can be associated with a single object, and these frames can be coincident or not (Figure 2).

**Figure 3:**
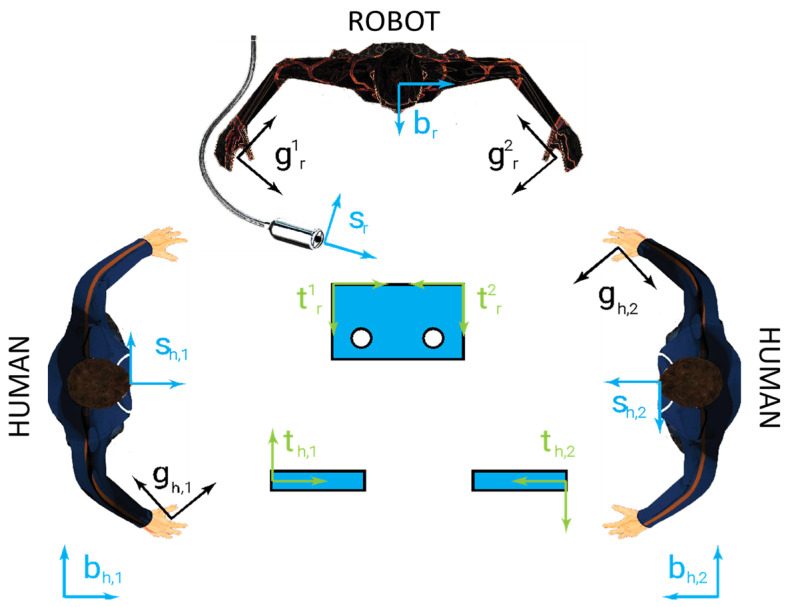
Reference frames in a collaborative layout between a human agent and a robot agent: ***s***, ***g***, ***t***, ***a***, and ***b*** are, respectively, the sensor, gripper, task, anti-task, and base reference frames.

Analogously, if different agents of the same type are considered, an integer subscript (starting from one) is associated with each agent, i.e., if two robotic agents are considered, the corresponding gripper reference frames are ***g_r,_*_1_** and ***g_r,_*_2_**. An example with two human agents and a single robot agent with two grippers is shown in Figure 3, where the anti-task reference frames are omitted for the sake of simplicity and are chosen in Formula (1) to limit the number of reference frames.

$$a_{h,1}^{1}\equiv t_{r}^{1}\;a_{h,1}^{2}\equiv t_{h,2}\;a_{h,1}^{3}\equiv g_{r}^{1}\;a_{h,1}^{4}\equiv g_{r}^{2}\;a_{h,1}^{5}\equiv g_{h,2}\;$$

(1) $$a_{h,2}^{1}\equiv t_{r}^{1}\;a_{h,2}^{2}\equiv t_{h,1}\;a_{h,2}^{3}\equiv g_{r}^{1}\;a_{h,2}^{4}\equiv g_{r}^{2}\;a_{h,2}^{5}\equiv g_{h,1}$$

$$a_{r}^{1,1}\equiv t_{h,1}\;a_{r}^{1,2}\equiv t_{h,2}\;a_{r}^{1,3}\equiv g_{r}^{2}\;a_{r}^{1,4}\equiv g_{h,1}\;a_{r}^{1,5}\equiv g_{h,2}$$

$$a_{r}^{2,1}\equiv t_{h,1}\;a_{r}^{2,2}\equiv t_{h,2}\;a_{r}^{2,3}\equiv g_{r}^{2}\;\;a_{r}^{2,4}\equiv g_{h,1}\;a_{r}^{2,5}\equiv g_{h,2}$$

If all the considered agents are of the same type or are not classified, the subscript r or h is omitted. Furthermore, if the dimensions of the objects are considered, the undesired contacts are not well defined only by the anti-task reference frame. The volume ***V*** of the points solid with the anti-task reference frame should be avoided by the volume ***V*** associated with the gripper reference frame. Furthermore, the task piece must also be gained only with the correct orientation, avoiding undesired contacts with this object; thus, an anti-task reference frame must be set on the task object and the volume ***V*** associated with the task object must be considered to correctly define the undesired contact. The superscript—is adopted in this last case to stress that there is a single task pose and a limited set of motion strategies that can be adopted to gain the task. Finally, the environment can be described as a whole single object with a reference system ***e*** and an associate volume ***V*** that must not be compenetrated. Thus, adopting a nomenclature similar to that of the anti-task references, in the presence of n agents with *m* grippers for each agent, the undesired contacts can be described by (2), where the volumes ***V****^j^_i_*(***g****^j^_i_*), ***V****^p^_q_*(***t****^p^_q_*), ***V*^−^**^,*j*^*_i_*(***t****^j^_i_*), and ***V***(***e***) are associated, respectively, with the *j*th gripper of the *i*th agent, to the *p*th target object of the *q*th agent, to the *j*th target object of the *i*th agent, and the environment.

$$V_{i}^{j}\left(g_{i}^{j}\right)\cap V_{q}^{p}\left(t_{q}^{p}\right)=\emptyset ;i=1\cdots n;j=1\cdots m;q=1\cdots n;p=1\cdots m;i\neq q;j\neq p$$

(2) $$V_{i}^{j}\left(g_{i}^{j}\right)\cap V_{q}^{p}\left(g_{q}^{p}\right)=\emptyset ;i=1\cdots n;j=1\cdots m;q=1\cdots n;p=1\cdots m;i\neq q;j\neq p\;$$

$$V_{i}^{j}\left(g_{i}^{j}\right)\cap V_{i}^{-,j}\left(t_{i}^{j}\right)=\emptyset ;i=1\cdots n;j=1\cdots m\;$$

$$V_{i}^{j}\left(g_{i}^{j}\right)\cap V\left(e\right)=\emptyset ;i=1\cdots n;j=1\cdots m$$

The grippers are moved by associated kinematic chains composed of different articulated bodies. This paper does not consider potential impacts between these kinematic chains; between the kinematic chains and grippers, targets, environment; or the internal bodies in each kinematic chain. In the following sections, some strategies are proposed to avoid the possibility of these undesired impacts.

## 3. The Proposed Approach

This section represents the theoretical innovation element of this paper and, after identifying a simple safety criterion in Section 4.1, proposes a topological treatment of relative transformations between references in Section 4.2 and a minimal but effective way of structuring the collaborative work cell in Section 4.3. Section 4.3 is a specification of the general treatment in view of the case study that will be expounded upon in Section 5.

### 3.1. The Proposed Topology Network of Reference Frames

The proposed approach allows considering only kinematical variables, while inertia and stiffness are approximately considered constant. This approximation allows to limit the computational intensity and has an approximated physical validity if the robot and other objects/agents can experiment with potential impacts only in a limited portion of the working space. Kinematical variables can be expressed in different reference frames, and each reference frame is related to the other reference frames through proper transformation matrices [38,39]. When the bodies are approximated as spheres with known radii, the center of each sphere with its associated radius can represent the whole sphere. Its position ***P*** can be represented by a set of homogeneous coordinates ***P****_i_*, with respect to the reference frame (*i*), with the notation in [38,39], and this set of coordinates ***P****_i_* is related to the coordinates ***P****_j_* concerning the reference frame (j) through the transformation matrix ***M****_i_*_,*j*_ (3).

(3) $$P_{i}=M_{i,j}P_{j}$$

The implementation of circumscribed spheres in the workspace facilitates the rapid computation of inter-surface distances, even in real-time scenarios, without necessitating precise identification of the complicated shapes of individual objects. Furthermore, the utilization of circumscribed spheres as a solution is deemed safe in terms of potential collisions between objects. This methodology is employed in scenarios where significant displacements are required to be executed expeditiously while simultaneously circumventing potential collisions. When two entities come into proximity and require interaction, the circumscribed sphere model is no longer appropriate. It is necessary to accurately determine the shapes of the objects involved and facilitate their interaction.

**Figure 4:**
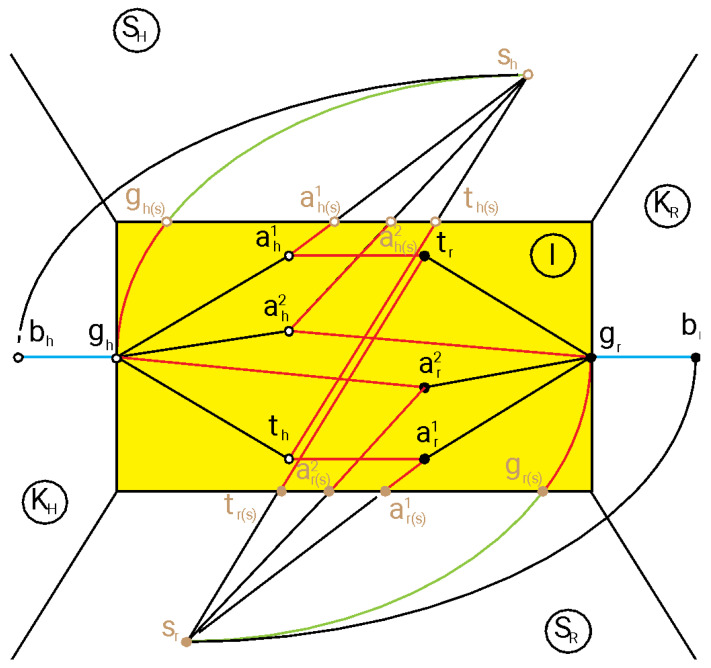
Example of the topological transformation network.

All the reference frames can be represented as a topological transformation network. Referring to the configuration depicted in Figure 2, a topological network with the principal reference frames and relative transformations is shown in Figure 4.

The topological space shown in Figure 4 is subdivided into five parts: K_H_ and K_R_ are the kinematical spaces, respectively, of the human and of the robot and are devoted to moving the grippers; ***S***_H_ and ***S***_R_ are the sensor spaces, respectively, of the human and of the robot and describe the perceptive space of each interacting agent; and I is the space of the interaction between the human and robot. Each node of the network represents a reference frame, and each connection between two nodes represents the transformation matrix between the two connected nodes. Arrows to indicate the directions of the transformations are not depicted to simplify the diagram. The black nodes are the same as described previously. The brown nodes represent the reference frame perceived by each sensor and the reference frame on each sensor, i.e., ***s***_r_ and ***s***_h_ are the reference frames, respectively, on the robot sensor and the human sensor; ***t***_r(s)_ and ***t***_h(s)_ are the target reference frames, respectively, perceived by the robot and by the human; ***g***_r(s)_ and ***g***_h(s)_ are the gripper reference frames, respectively, perceived by the robot and by the human; and ***a***^i^_r(s)_ and ***a***^i^_h(s)_ are the *i*th anti-target reference frames, respectively, perceived by the robot and by the human. The empty nodes are associated with the human, whereas the full nodes are associated with the robot. The blue lines represent a kinematic chain from the base node to the gripper node. In the context of this work, they are serial kinematic chains of rigid bodies and thus constituted by a product of the relative transformation matrices. The red lines represent changes of the reference frames on the same body, i.e., all the reference frames attached by red lines are attached to the same rigid body. The black continuous lines represent transformations between the interacting bodies. The dashed black lines represent transformations between perceptive reference frames and sensor frames in their respective perceptive sensor spaces. In general, perceptive reference frames are not coincident with objective reference frames in the interaction space I. The dashed green lines represent the proprioceptive transformations between the gripper and sensor frame of each agent. The dash–dot black lines represent the objective transformations between the base and the sensor frame of each agent; in this work, it is supposed that these transformations can be realized with an articulated serial kinematic chain or with a fixed constraint.

Let us focus the attention on the robot viewpoint. Black continuous transformations are associated with safety criteria, because they describe the geometrical relations between the gripper and other objects in the interaction space; furthermore, the transformation between the gripper and associated target also has a functional characteristic, because it describes the action requested of the gripper. Reference frames associated with objects in the interaction space that do not belong to the robot are perceived by the robot sensor passing through the associated transformations described by the dashed black lines. The proprioceptive transformation is not generally necessary during the interaction but can be used to autocalibrate the robot; in fact, the gripper is moved by the robot kinematic chain with its internal real-time sensors, and its pose is known by the robot. The transformation between the sensor and the base of the robot is considered known. In general, the robot can know the human sensor frame and the human base frame if it knows the human kinematic chain and structure. Some alternative approaches can be adopted to know the human sensor frame, directly identifying it. This knowledge can be useful to implement the theory of mind and to realize comfortable movements. If each transformation is an identity, each corresponding line degenerates to a single point. Furthermore, each sensor can be connected to a body of the kinematic chain that connects the base to the gripper or directly to the gripper or other bodies in the interaction space I.

The safety variable can be associated, as just mentioned, with the transformations from the gripper to the corresponding anti-targets, as shown in (4), where it is assumed ***a****^n^_r_* is equal to ***t****_r_*, ***P****^i^_r_* is a point of the anti-target *i* described in the reference frame ***a****^i^_r_*, and ***P****_g_* is the same point described in the reference frame ***g****_r_*.

(4) $$P_{r}^{i}=M_{i,g}P_{g},\;i=1,2\cdots n\;$$

Under the approximation of interacting bodies as spheres, only the translational term ***T****_i_*_,*g*_ of the matrix ***M****_i_*_,*g*_ expressed in (4) is necessary to describe the vector ***d****_i_*_,*g*_ of the minimal distance between the anti-target *i* and the gripper ***g****_r_*; then, the measure of the distance (3) can be computed with a proper measure function, for example, the Euclidean distance (5).

(5) $$d_{i,g}=T_{i,g}-R_{i}-R_{j},\;T_{i,g}=\sqrt{T_{x\;i,g}+T_{y\;i,g}+T_{z\;i,g}}$$

The target function can be associated, analogously, with the transformation ***M****_t_*_,*g*_ between the target and the gripper, as shown in (6), where ***P****_t_* is a point of the target *t* described in the reference frame ***a****_t_*; ***P****_g_* is the same point described in the reference frame ***g****_r_*.

(6) $$P_{t}=M_{t,g}P_{g}$$

In this case, the gripper and the target must be at a correct distance with the correct orientation; thus, a more complex distance function must be adopted [40,41]. In general, if the desired relative orientation is ***M****^d^_t_*_,*g*_, a proper motion planning of the gripper must be implemented to reduce the measure ε of the error matrix **∑** shown in (7).

(7) $$\Sigma =M_{t,g}^{d}-M_{t,g}$$

If the computations are referred to the base reference frame ***b****_r_*, the transformation matrix ***M****_i_*_,*g*_ can be expressed as in (8) with a matrix product between the pose of the ith anti-target ***M****_i_*_,*b*_ and the known pose of the gripper ***M****_b_*_,*g*_; then, Expressions (5) and (7) can be adopted, respectively, to compute the distance between the gripper and the anti-target and to monitor the realization of the target.

(8) $$M_{i,g}=M_{i,b}M_{g,b}^{-1}=M_{i,b}M_{b,g}$$

Furthermore, the pose of the *i*th anti-target and the target are known through the sensor ***s***_r_, and the relative pose between the sensor and the base ***b****_r_* is known; thus, the matrix ***M****_i_*_,*g*_ can be referred to the reference frame ***s***_r_, as shown in (9).

(9) $$M_{i,g}=M_{i,s}M_{g,s}^{-1}=M_{i,s}\left(M_{s,b}M_{b,g}\right)$$

Comparing Expressions (4), (8) and (9), it appears that using a single reference frame centered on the gripper can lead to a computational reduction for the safety indices, but the poses of the target and anti-target objects are known in the sensor reference frame. Thus, a possible solution to reduce the computational intensity by adopting directly measured data is locating the sensor on the gripper. The problem with this approach is associated with the reduced visibility of the interaction space; thus, its adoption is generally limited in the literature, except for touch sensors [42], and it is certainly an interesting research field. In this work, the principal robot sensor will be fixed on the base reference frame. An alternative solution is locating the robot sensor reference frame corresponding to the human sensor reference frame by adopting sensorized glasses. This solution leads to the possibility of the theory of mind for the robot, because it can perceive the interaction environment from the human viewpoint, incrementing human comfort.

Along the kinematic chain, different local reference frames are adopted to define the relative motion between the bodies of the chain and associated motor movements, as is widely known in robotic kinematics.

Kinematic computations can be realized in a parallel way by adopting simultaneously different reference frames, but, finally, the decision on the gripper movement is one; thus, all these computations must be joined into a single processing unit.

### 3.2. The Proposed Procedure for Layout Arrangement

The robot activity can be subdivided in three functional steps: the input of raw pieces, working activity, and output of worked pieces. The interaction occurs in one or more of these areas, as shown in Table 2, where the column “Interactions” summarizes the number of areas where the interaction can occur.

Focusing the attention on a single robotic agent with a single gripper, the steps shown in Table 2 can be considered always sequential from the robotic agent viewpoint; thus, any eventual complex working activity can be subdivided into a sequence of simple activities, where the internal input and output steps separate each working activity from the others. This approach can also be, in general, adopted to separate activities in a way that only cases (a), (b), and (c) listed in Table 2 can occur. Then, we suppose to associate separated physical areas with separated functional steps; in this way, an eventual interaction occurs in an identified area with a single type of risk [9,43].

According to ISO/TS 15066 [44], contacts between the robot and specific parts of the human body must always be avoided, i.e., head, genitals, and breasts; furthermore, contact with the principal hand of the human should be as limited as possible [45]; finally, other undesired contacts should be limited. To respect these constraints, the layout of the system is structured with these precautions, if it is possible:

I. The robot is positioned as far as possible from the human agent;
II. The functional activities occur on a planar desk over the genital level and parallel to the transverse (i.e., horizontal) plane of the human agent;
III. The robot is positioned at the side of the secondary hand of the human;
IV. A virtual barrier is positioned between the robot and the head/breasts of the human, with a passage under these levels;
V. The kinematic chain of the robot must be always farther from the human agent than its gripper;
VI. The gripping and working actions are realized in a plane parallel to the transverse (i.e., horizontal) plane of the human agent;
VII. Only necessary objects are present in the area of interest.

**Figure 5:**
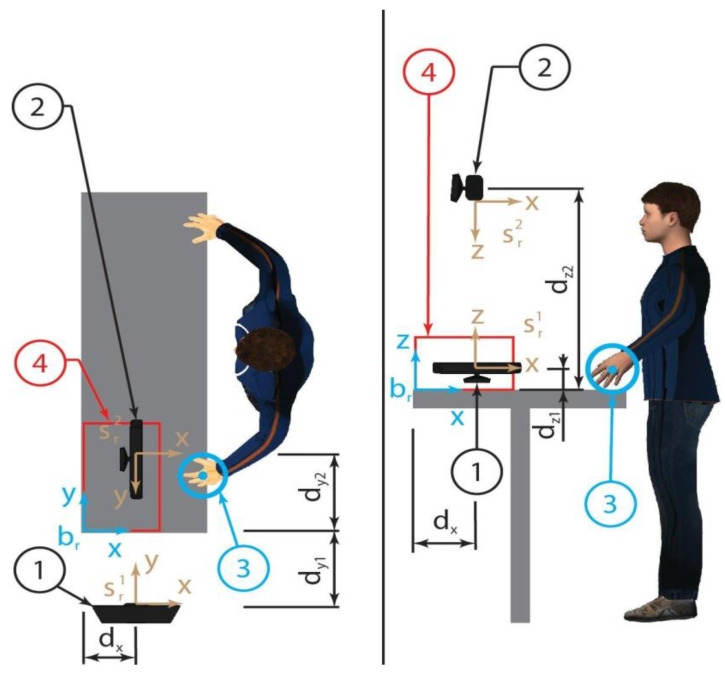
Robot sensors 1 and 2 fixed to the reference frame, and observing the interacting hand 3 when it is in the interaction area 4.

If it is possible, one or more of these constraints, barriers, or motion strategies can also be set virtually, i.e., within the motion planning algorithm. When the barriers are virtual, the position and velocity of the human agent must be monitored, or some other safety precautions must be implemented to limit damage to the human agent. Constraint (I) allows to limit the intersection between the robot and the human working spaces, thus limiting the interaction area and the probability of undesired impacts; furthermore, the human can interact with the robot only with one or two hands, while other parts of the body cannot enter the interaction area. Constraint (II) does not allow impacts with the genitals, limits impacts with the head, reduces possible interactions between human and robot agents, and simplifies the identification of targets and anti-targets. When it is possible, constraint (III) limits impacts with the principal hand of the human agent; furthermore, the secondary arm can be used as a shield in case of an emergency. Constraint (IV) does not allow impact with the head, limits impact with the breasts, and maintains the robot kinematic chains behind the gripper. Constraint (V), when implemented in a motion planning algorithm, maintains the robot kinematic chains behind the gripper, limiting impacts between the human and the arm of the robot. Constraint (VI) reduces the interactions between robot gripper and human and between the robot arm and human with a negative mechanical effect on the robot working step. In fact, the reaction of the target object to the robot action can be transferred to the environment only through the friction between the piece and the planar desk. Thus, if it is necessary, an increment of the friction can be realized with a pad or with a vertical reaction surface fixed to the planar desk, which can also be used to limit human–robot impacts and to constrain the position of the target object. Constraint (VII) highly accelerates the object identification process and limits eventual impacts. With this scenario, the human agent has to adopt a hand pose with the back oriented in the up/external directions; thus, two sensors fixed to the base of the robot are adopted: one from up to down and the other from the external to the internal direction of the principal hand of the human agent (Figure 5).

In the layout shown in Figure 5, the positions of the sensors are known with respect to the absolute reference frame ***b****_r_* through the parameters ***d****_x_*, ***d****_y_*_1_, ***d****_y_*_2_, ***d****_z_*_1_, and ***d****_z_*_2_. The absolute reference frame is fixed to the base of the robot and is known after a preliminary calibration. The sensor reference frames ***s***_r1_ and ***s***_r2_ are oriented with respect to the absolute reference frame ***b****_r_* through translations and rotations of π/2, π, 3π/2, or 2π to simplify the transformation of the respective coordinates. Thus, the position of the hand marker, corresponding to the center of sphere 3 in Figure 5, is described by the coordinates corresponding to the absolute reference frame (subscript 0) related to the coordinates with respect to sensor frame 1 (subscript 1) and to sensor frame 2 (subscript 2) according to Expression (10), where ***ε****_xi_*, ***ε****_yi_*, and ***ε****_zi_* are the measurement errors of the perceived coordinates ***x***, ***y***, and ***z***, respectively, by the sensor *i*.

$$x_{0}=d_{x}+x_{1}+\varepsilon_{x1}=d_{x}+x_{2}+\varepsilon_{x2}$$

(10) $$y_{0}=y_{1}-d_{y1}+\varepsilon_{y1}=d_{y2}-y_{2}+\varepsilon_{y2}$$

$$z_{0}=d_{z1}+z_{1}+\varepsilon_{z1}=d_{z2}-z_{2}+\varepsilon_{z2}$$

The interaction area is identified by a parallelepiped (red and marked with the number 4 in Figure 5) with dimensions ***D****_x_*, ***D****_y_*_,_ and ***D****_z_*. When the hand is outside the interaction area, the position of the marker is set conventionally, as in (11), where ***R****_h_* is the radius of the approximated sphere circumscribed to the hand; ***v****_h_* and ***a****_h_* are, respectively, the conventional hand speeds and acceleration.

$$x_{0}=D_{x}+R_{h},\;y_{0}=D_{y},\;z_{0}=R_{h}\;$$

(11) $${\dot{x}}_{0}=v_{h},\;{\dot{y}}_{0}=v_{h},\;{\dot{z}}_{0}=v_{h}$$

$${\ddot{x}}_{0}=a_{h},\;{\ddot{y}}_{0}=a_{h},\;{\ddot{z}}_{0}=a_{h}$$

The Equation (11) is a limit equation with the approximated sphere circumscribed to the hand tangent externally to the interaction a. When the marker on the back of the hand is perceived by a sensor, its position is set according to (10), while the velocity and acceleration are computed by taking into consideration the time interval Δ***t*** between two observations, as shown in (12), for the time instant ***t****_i_*, supposing that the presence of the hand in the sensing area (generally greater than the interacting area) started before the time instant ***t****_i_*_−1_. Eventually, proper filters can be adopted if high time–frequency errors are present to limit the effects of numerical differentiation.

(12) $$\begin{array}{c} {\dot{x}}_{0}\left(t_{i}\right)≅\frac{\left[x_{0}\left(t_{i}\right)-x_{0}\left(t_{i-1}\right)\right]}{\Delta t},\;{\dot{y}}_{0}\left(t_{i}\right)≅\frac{\left[y_{0}\left(t_{i}\right)-y_{0}\left(t_{i-1}\right)\right]}{\Delta t},\\ {\dot{z}}_{0}\left(t_{i}\right)≅\frac{\left[z_{0}\left(t_{i}\right)-z_{0}\left(t_{i-1}\right)\right]}{\Delta t}\\ {\ddot{x}}_{0}\left(t_{i}\right)≅\frac{\left[{\dot{x}}_{0}\left(t_{i}\right)-{\dot{x}}_{0}\left(t_{i-1}\right)\right]}{\Delta t},\;{\ddot{y}}_{0}\left(t_{i}\right)=\frac{\left[{\dot{y}}_{0}\left(t_{i}\right)-{\dot{y}}_{0}\left(t_{i-1}\right)\right]}{\Delta t},\\ {\ddot{z}}_{0}\left(t_{i}\right)=\frac{\left[{\dot{z}}_{0}\left(t_{i}\right)-{\dot{z}}_{0}\left(t_{i-1}\right)\right]}{\Delta t} \end{array}$$

If the hand marker is observed by both sensors, the mean value of the two observations described in (10) can be adopted to limit the measurement errors, as shown in (13).

$$x_{0}≅d_{x}+\left(x_{1}+x_{2}\right)/2$$

(13) $$y_{0}≅\left(y_{1}-y_{2}\right)/2+\left(d_{y2}-d_{y1}\right)/2$$

$$z_{0}≅\left(z_{1}-z_{2}\right)/2+\left(d_{z1}+d_{z2}\right)/2$$

**Figure 6:**
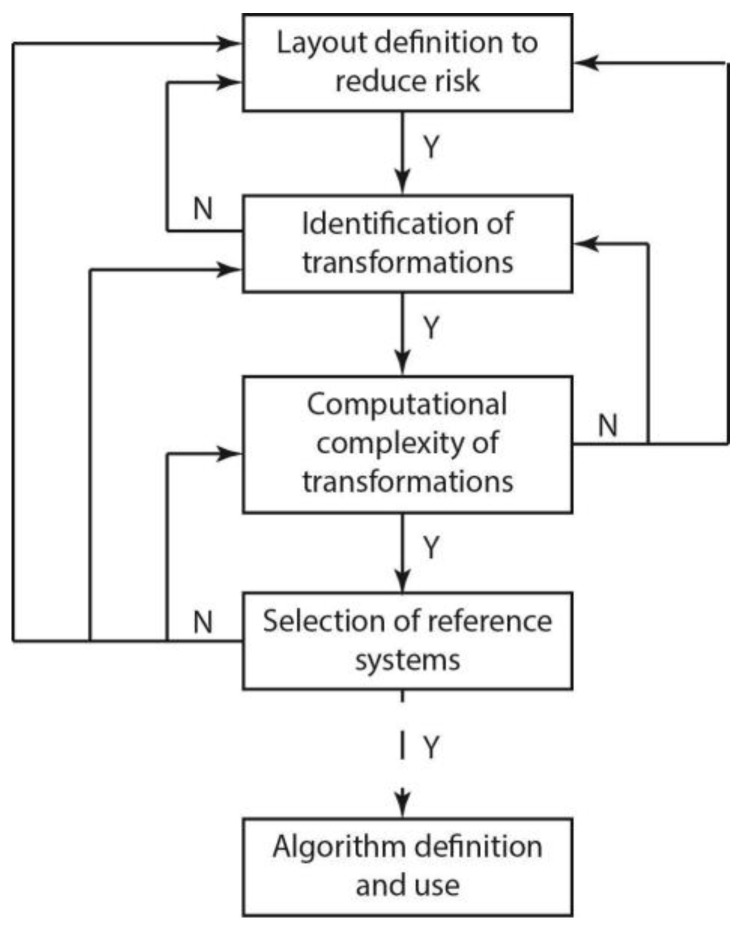
Process flowchart.

**Figure 7:**
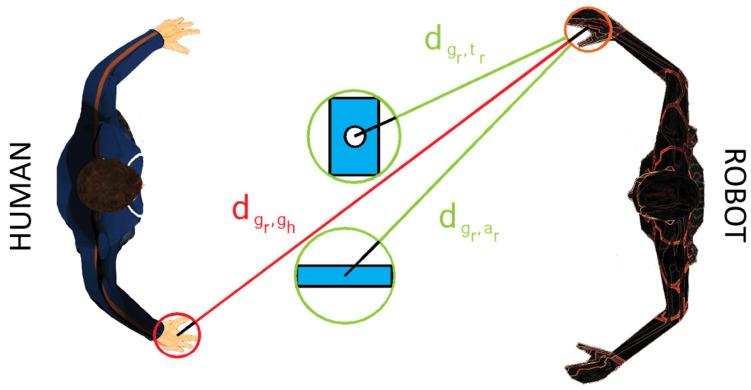
Distance between circumscribed spheres with known radii.

If an object is observed by one or both sensors and it is not recognized as a target, an anti-target, or the robot gripper, it is interpreted as a human hand, although the hand marker is not observed by one or both sensors. Part of the approximated sphere associated with the human hand is reconstructed with the following fast algorithm to allow a rapid definition of the motion strategy for the robot gripper. Figure 6 represents a flowchart to clarify the whole process. Furthermore, also, if the information on the position of the robot gripper from the robot kinematic chain is not considered, the human hand cannot be confused with the robot gripper, because they enter the interaction area from different sides, as shown in Figure 5 and Figure 7.

In this work, in order to track the operator’s movements, instead of using an instrumented glove or two camera sensors, an open-source, pretrained convolutional neural network was employed, taking advantage of a single-depth camera. This solution allows the identification of the characteristic points of the person’s skeleton, i.e., the position of the body joints, by exploiting the RGB frame provided by the camera. By combining the position of the skeleton points within the color frame and the distance information contained in the depth frame acquired by the camera, it is possible to reconstruct the skeleton in a three-dimensional space.

### 3.3. Quantitative Measures of Safety

Expression (2) means that the considered grippers must maintain a certain distance from anti-task bodies. This distance can be, in general, different for different categories of bodies for safety reasons; in particular, a higher safety distance must be considered from human bodies than that from inanimate bodies, because impacts involving human bodies can produce physical damage to humans. The concept of distance is an open research field, and different definitions can be adopted [40,41]. To reduce the risk of impacts and limit the computation intensity, a simplification is proposed in this work considering circumscribed spheres in a collaborative system, and this solution allows to neglect the orientation of bodies. The association of points to each object can be, in general, realized with artificial vision techniques [46]; then, sphere identification can be easily implemented as the smallest circle problem, which can be parallelized. Finally, the distances between objects *j* and *k* can be computed as the distance ***d****_j_*_,*k*_ between the two spheres *j* and *k*, as described in (14), where ***x****_j_*_,*i*_ and ***x****_k_*_,*i*_ are, respectively, the coordinates of the centers of spheres *j* and *k*; ***R****_j_* and ***R****_k_* are, respectively, the radii of spheres *j* and *k*.

(14) $$d_{j,k}=\sqrt{\overset{3}{\underset{i=1}{\sum }}{\left(x_{j,i}-x_{k,i}\right)}^{2}}-R_{j}-R_{k}$$

If a dimension of an object is dominant, the circumscribed sphere can occupy an excessive volume; thus, a different solution can be adopted approximating the object with two or more adjacent spheres along a dominant rectilinear or curved line. An analogous solution can be adopted when two dimensions of an object are dominant; in this case, the object can be approximated with three or more adjacent spheres along a dominant planar or curved surface. Alternatively, sphere packing techniques can be adopted [47]. If the working area contemplates the presence of only human/robotic grippers and pieces to be assembled or worked, the radii of the associated spheres can be considered as known parameters, reducing, in this way, the computational intensity (Figure 7). As will be shown in the next sections, a proper definition of the system layout is sufficient to allow this simplified condition.

As widely known, in the presence of an eventual impact, the kinetic energy of impacting bodies can be transformed into deformation energy-producing damages; thus, to measure the possibility of damages, inertial characteristics and relative speeds are physical quantities that must be monitored to limit the safety risks. The impact phenomena do not allow neglecting all the kinematic chain and base constraints of the robot and human agents, where constraint reactions exhibit impulsive values. The sphere shape approximation introduced can simplify the impulsive equilibrium of the impacting bodies. A hypothesis of a completely inelastic impact between human and robot bodies, such as a completely elastic impact between robots and other inanimate bodies, can surely simplify the dynamic analysis of the system. To reduce the computational intensity even more, this work proposes to consider impact criteria adopted in the literature to preserve the integrity of the human head. These criteria are characterized by fixed inertia parameters of the robot and the human, by a fixed stiffness parameter of the robot, and by a relative variable speed of the gripper. The objective is to limit the speed to minimize the human damage associated with the transformation of kinetic energy into deformation energy. In [48,49], the authors showed that a safety value (HIC) depends on the velocity *v* through a constant value α that incorporates the inertia and stiffness (15). The constant parameter α can be easily computed with information from the datasheet of the robot and from the biomechanical characteristics of the human agent, as shown in (16), where *g* is the gravitational acceleration, *m_h_* is the mass of the human head, *m_r_* is the moving robot mass of the robot, *p* is the payload, and *k* is a combined stiffness between the robot and head of the human agent. The combined stiffness depends on the robot stiffness *k_r_* and the head stiffness *k_h_* (17).

(15) $$HIC_{simplified}=\alpha \cdot v^{5/2}$$

(16) $$\alpha \approx 1.40\cdot \pi {\left(\frac{1}{2\cdot g}\right)}^{\frac{5}{2}}{\left(\frac{k}{m_{h}}\right)}^{\frac{3}{4}}{\left(\frac{m_{r}+p}{m_{r}+p+m_{h}}\right)}^{\frac{7}{4}}$$

(17) $$k=\frac{k_{r}k_{h}}{k_{r}+k_{h}}$$

HIC is used to set a limit for the velocity v of the robot when it can impact a human; instead, this work uses this expression for a different purpose. In this work, the speed of the gripper or, eventually, the relative speed between the gripper and other objects can be monitored in real time, and when the distance between the object and the gripper is under a safety value, the speed can be limited. This distance depends on different characteristics of the system, and the motion strategy will be described in the next sections after the introduction of some assumptions on the system layout.

## 4. A Case of Study of Human–Robot Collaboration

### 4.1. The Proposed Experimental Set Up

The experimental setup on which the developed framework was tested is a collaborative robotic cell located in the laboratory of Smart Automation and Robotics (SAR) of the University of Brescia.

**Figure 8:**
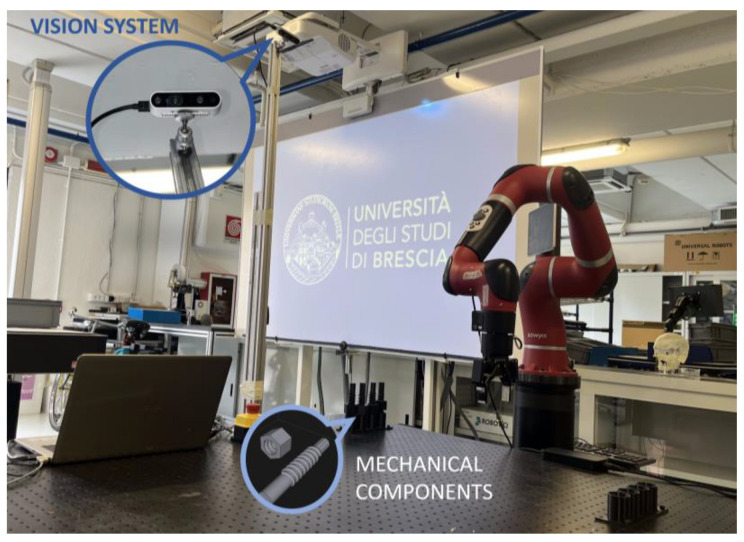
Experimental setup consisting of a Sawyer cobot, vision system (RealSense D435 camera), and the mechanical tools highlighted.

Figure 8 shows the robot used in the application and the camera that monitors the collaborative space. The developed software runs on a low-cost workstation equipped with an Intel 8750 CPU and an NVIDIA GeForce GTX 1050 GPU. Specifically, for data and frame acquisition, both the color and depth frames are managed in the ROS environment. The robot used in the experiment is the model Sawyer manufactured by Rethink Robotics. It is a seven-degree-of-freedom (DOF) anthropomorphic manipulator that is included in the category of collaborative robots because it can work in direct and safe contact with a human operator. Sawyer is classified in the category of “power and force limited by inherent design” [50]. The payload of the robot is 4 kg, and it has a nominal repeatability of ±0.1 mm. There are protective elements of soft material on the joints to increase safety, and it is also equipped with both electrical and mechanical brakes, which, combined with torque sensors on each joint, allow the motion to stop in case of accidental impact. The depth camera used in the experiment is a RealSense D435, and it is equipped with a color sensor (RGB module) and a module dedicated to the stereo depth vision. The dedicated depth vision module consists of two sensors (named Right and Left Imager), an infrared projector to illuminate the scene, and a Vision Processor. The depth frame is reproduced using stereoscopic vision. From the correspondence of the images produced by the left and right sensors, the D4 processor is able to calculate the disparity, i.e., the shift of the same pixels in the two images, calculating the distance and, thus, constructing the depth map. In this experiment, the camera was placed at the side of the robotic cell at a height of 2 m from the working area. The orientation of the camera was chosen to appropriately frame the collaborative space shared between the robot and the operator. In this way, it is possible to monitor the person’s movements when approaching the robot. A key aspect of the application is to identify the operator key points (i.e., the body joints of the skeleton present in the image detected by the camera) framed by the camera in a real-time and reliable way. Over the years, several algorithms have been developed to perform pose estimations of people within an image [51]. Among several possibilities, MediaPipe Pose was chosen [52], which is a lightweight convolutional neural network architecture used for real-time and high-fidelity body pose tracking, inferring 33 3D landmarks, and background segmentation masks on the whole body from RGB frames.

### 4.2. Implementation of the Proposed Procedure

**Figure 9:**
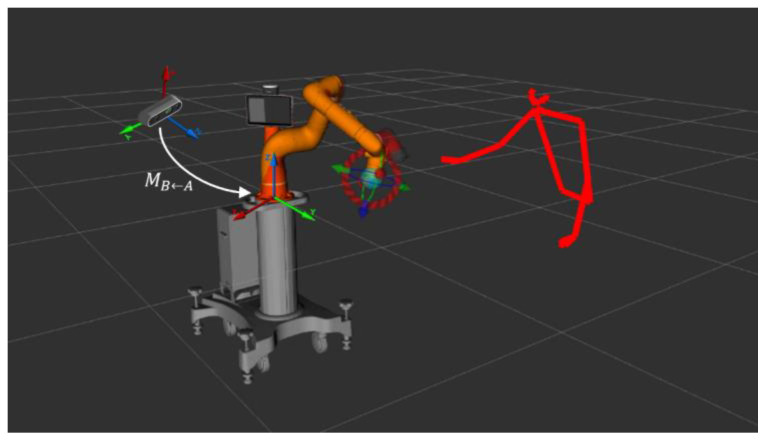
System reference frame and skeleton reconstruction.

The main objective of this paper is to discuss the human–robot interaction and the strategy for reference frame identification. In order to evaluate the safety assurance, it was necessary to realize a workstation where the robot’s and the operator’s frames overlapped during the execution of a task. The realized application consists of a simple human–robot collaboration. Specifically, the machine is in charge of performing the choice and place of mechanical components made through additive manufacturing (visible in Figure 8), and then, the human operator is asked to perform a screwing operation on the components carried by the robot. The area where the screws are released corresponds to the operator’s workspace, which could lead to a collision of the two entities without the implementation of safety measures. A key aspect in the formulation of the problem is the definition of a global reference system against which the key points of the person’s skeleton can be referenced. Figure 9 shows the reference frame of the robot and the camera reference system against which the key points of the skeleton are calculated. The skeleton reconstruction and filtering algorithm produces the information in the camera reference system. Then, the points are converted into the robot reference system.

The transformation matrix ***M***_Robot_,_Camera_ denotes the position of the camera reference system relative to the robot’s base reference system; this transformation matrix is defined through a calibration procedure called hand-eye (eye-to-hand version) [53,54].

Figure 9 also shows the results of the person’s movement tracking algorithm. Through the identification of the key points of the skeleton, it is possible to read within the depth map the relative distance between them and reconstruct the skeleton in the camera reference system. In order to reduce the jerky behavior associated with the depth map information, the position of each key point is filtered using a Kalman filter in order to smooth the distance information. Once these points have been filtered, the skeleton referenced in the robot reference system is generated so that the human–robot distance is known in real time based on the equation explained before in Section 2. The developed tracking, skeleton reconstruction, and filtering algorithm are then combined with an algorithm to modulate the robot’s speed based on the human–robot distance. The ISO 15066 technical standard defines four different scenarios of human–robot interactions: SMS, HG, SSM, and PFL (refer to [44,55]). The “Speed and Separation Monitoring” (SSM) mode appears to be the most efficient and flexible for general-purpose collaborative applications. It is in this replanning approach that the information produced by skeleton reconstruction developed in the work finds use. The minimum separation distance must take into account the space covered by the robot and the operator within the reaction time of the robotic system, the stopping distance required by the robot, and the position uncertainty of the operator and robot in the collaborative space. The following expression can be formulated to calculate the separation distance minimum required. Synthesizing these specifications, starting from the definition of the minimum separation distance given in ISO/TS 15066, it is possible to reformulate this distance as follows:

(18) $$S_{p}=v_{h}\left(T_{r}+\frac{v_{r}}{a_{s}}\right)+v_{r}T_{r}+\frac{v_{r}}{2a_{s}}+C<S\;\left(t_{0}\right)$$

where

- $v_{h}$ is the speed of the operator in the direction of the robot (in case it is not detected by the motion tracking system, the ISO/TS 15066 suggests using $1.6\;\frac{m}{s}$ as the reference value in the direction of separation distance reduction to be conservative);
- $v_{r}$ is the speed of the robot in the direction of the person;
- $T_{r}$ is the reaction time of the robot, which includes the time taken by the tracking system to detect the position of the operator until the activation of the robot’s stop signal;
- $a_{s}$ is the maximum deceleration of the robot to stop the motion;
- C is the parameter that takes into account the detection uncertainty of the position of the person and the robot;
- $S\left(t_{0}\right)$ is the robot–operator distance at any time $t_{0}$.

The distance of the robot from any part of the operator’s body should be greater than the minimum required separation distance. From Equation (18), it follows that the maximum speed for the robot must be consistently maintained below

(19) $$V_{r,max}=\sqrt{v_{h}{}^{2}+{\left(a_{s}T_{r}\right)}^{2}-2a_{s}\left(C-S\left(t_{0}\right)\right)}-a_{s}T_{r}-v_{h}\;$$

**Figure 10:**
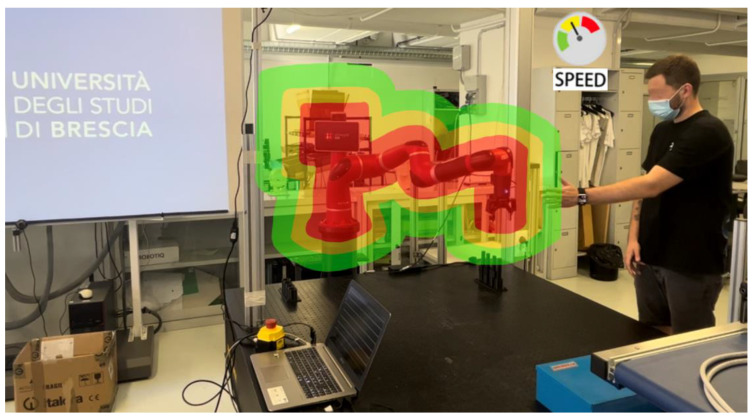
Example of the implemented scaling algorithm; based on the minimum separation distance calculated by Equation (5), the maximum speed of the robot is modulated with override from 100% (green zone) down to 0% (full stop, red zone).

In summary, Equation (18) can be used to calculate the minimum separation distance between the spheres generated by the anti-targets defined in Section 4. In addition, Equation (19) defines the velocity scaling of the robot as the distance between the anti-targets varies. To simplify the computational time of the chosen scaling algorithm, it was decided to divide the maximum speed executable by the robot into three different areas based on the minimum separation distance, as shown in Figure 10. When using a collaborative robot in a standard scenario, safety countermeasures are only triggered by an impact, whereas, with this method, contact between the human and robot can be avoided through speed reduction. Additionally, to ensure robustness in human pose 3D reconstruction, considering potential occlusions or complex poses, a sensor fusion solution using multiple cameras can be adopted, as proposed in [56,57,58], while maintaining the overall approach.

### 4.3. Results of the Experimental Tests

**Figure 11:**
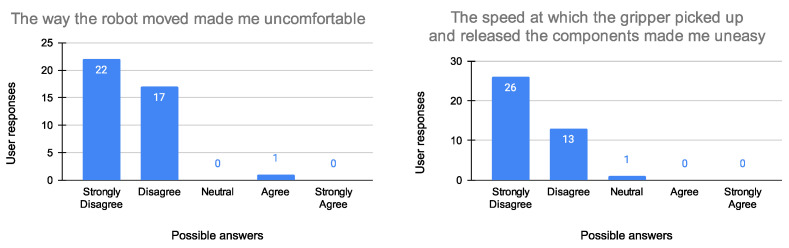
Evaluation of the Robot’s motion and pick-up speed (Question 1 on the (**left**), Question 2 on the (**right**)).

**Figure 12:**
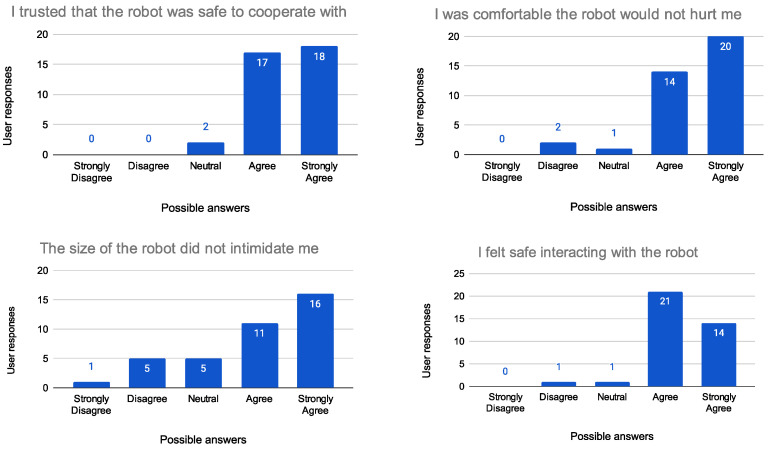
Evaluation of the Safe cooperation (Question 3 on the (**top left**), Question 4 on the (**top right**), Question 5 on the (**bottom left**), and Question 6 on the (**bottom right**)).

**Figure 13:**
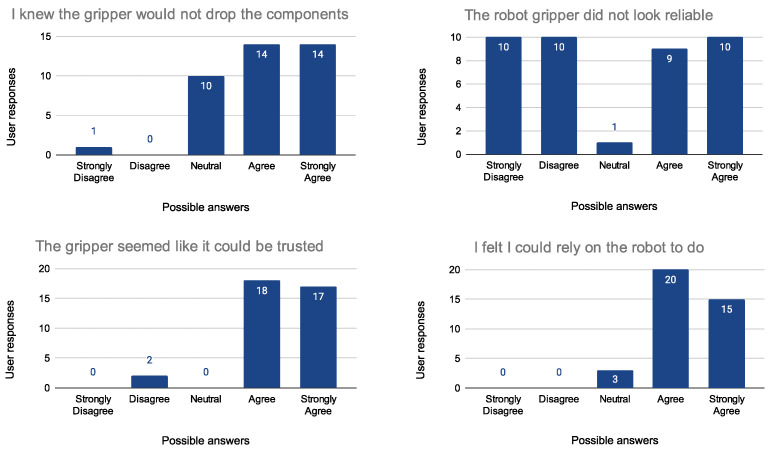
Evaluation of the Robot and gripper reliability (Question 7 on the (**top left**), Question 8 on the (**top right**), Question 9 on the (**bottom left**), and Question 10 on the (**bottom right**)).

To evaluate the proposed system, experiments have been carried out to evaluate the user’s experience with people of different ages, sex, and professional backgrounds. Trust and safety have been identified as key elements for successful cooperation between humans and robots. Although trust has received extensive attention in the last years, little research has focused on understanding trust development in human–robot collaborations. To appropriately understand the development of trust between human workers and robots, the author of [59] realized a measurement tool that offers the opportunity for system designers to identify the key system aspects that can be manipulated to optimize trust in HRC. The aim of the study was to develop an empirically determined psychometric scale to measure trust in HRC. There are three key factors (components), each of which is assessed with several items, to make people feel comfortable operating alongside robots. The first component is termed “Safe co-operation” and consists of four items, component 2 is termed “Robot and gripper reliability”, which consists of four items, while component 3 is termed “Robot’s motion and pick-up speed”, consisting of two items, as explained in Table 3. Based on this knowledge, the people involved in the experiment were asked to fill out a questionnaire to assess the effectiveness of the collaboration between them and the robot after performing the task explained in Section 4. The questionnaire required them to rate 10 statements (see Table 3) on a five-point scale ranging from Strongly Disagree to Strongly Agree. A total of 40 subjects were tested, equally divided between males and females and subdivided into two age groups (ages 20–30: 27 subjects and 30–65: 13 subjects). Of all the subjects interviewed, only four said they were already familiar with using robots; all others were treated as “inexperienced” users. The results of the survey are presented in Figure 11, Figure 12 and Figure 13.

As highlighted by the surveys, people were generally satisfied with carrying out the collaboration task and approached it with enthusiasm, also demonstrating a high level of trust associated with the workstation. Dividing the results into the three major components of the confidence scale, it is possible to say that, regardless of age, gender, or professional background, all the people felt more or less comfortable working with the robot. It is worth noting that the only uncertain parameter concerned the “Robot and gripper reliability” category. Almost half of the respondents said that the gripper did not seem reliable in contrast to the results obtained for the question “The gripper seemed like it could be trusted”. Participants were then asked for explanations, and all of them said that they were initially worried that the gripper might crush their fingers while gripping, but after taking the test, they felt safe, as the robot did not come close enough to their hands during the execution of the task.

## 5. Discussion

The development of an experimental platform for testing collaborative techniques and algorithms to increase the safety and comfort of the human agent without reducing the overall efficiency of the work process was enabled by a review of the literature and the formulation of a scheme for the realization of a collaborative configuration, which included the robotic agent, human agent, workpiece, environment, and sensors. The experiment involved executing a collaborative task that required physical proximity between the robotic agent and the human. The presentation of a psychometric test to the human agent demonstrated a good degree of comfort, and no mishaps happened during any of the procedures. The scientific literature addresses the comfort issue in the human–robot relationship in three broad ways: analyzing the human agent’s psychological representation of the robot and the environment, developing movements that prevent the robot from getting too close to the operator or moving at the boundaries (or even outside) of the human agent’s cone of vision, and monitoring the operator’s posture with sensors. The literature research focused on the operator’s relative position in relation to a mobile robot that followed the human subject’s movements but did not create obstructions [60]. Other authors emphasized the visibility of the robot’s moving elements as a factor that contributes to comfort [61]. Changizi and Lenz [62] expanded the comfort zone feature by defining two major categories: bodily comfort and mental comfort based on these kinematic techniques. This second category extended the human–robot comfort notion to the sciences of psychology and cognitive science, making Changizi and Lenz’s work an intriguing multidisciplinary connecting point. Human–subject comfort can then be measured through methods used in psychology, such as surveys, interviews, or observations by expert subjects, or objective neurological examination [63]. The approach described in this research, on the other hand, concentrated on the robot’s posture as the chosen aspect and then applied all of the previously proposed and listed strategies concurrently in the literature. In fact, after developing a representative model of the human–robot relationship, a set of rules was proposed in Section 3.2 to allow the robot to configure itself into the optimal posture, followed by the implementation of a sensorized layout, safe and comfortable movement strategies, and finally, the psychological state of the human agent was measured. The acquired results agreed with the literature in terms of the kinematic, position, and relative velocity aspects; used measurement methods based on tests provided to the operator; and introduced some unique aspects of work cell structuring, including the use of virtual instrumentation. The measured comfort of the human–robot relationship demonstrated that the method adopted was mainly adequate. Finally, the set of guidelines proposed in Section 3 and the associated analytical–topological treatment enabled the development of a safe and comfortable approach to the human–robot relationship with satisfactory experimental results when compared to previous studies.

Multiple lines of research may represent elements of improving the interaction abilities of collaborative robots. The latest algorithms in the literature are expanding the possibilities of object recognition in the presence of little information, for example, with a limited number of shots [64]; this line of research could allow the complexity of the workspace to be expanded by allowing less structuring of the environment. In certain cases, new intelligent algorithms allow interactions with unknown objects, seizing their application opportunities in the work context [65]. In these cases, the flexibility of grippers capable of manipulating objects in different grasping modes is a factor in enriching the robot’s potential [66]. Furthermore, an underexplored topic pertains to the delineation of the environment, which, within the scope of this study, manifests as a passive domain wherein human and robotic agents engage in an interaction. The enhancement of environmental awareness can be achieved through the utilization of sensors placed in hazardous areas with a tactile nature [67] or obtained from other domains, such as biomedical engineering, where the acquisition of biokinematic or biodynamic data is commonplace [68]. This approach enables the amplification of local information to facilitate the implementation of active environmental elements that can provide feedback, such as vibratory signals [69] to indicate danger, or passive interdiction of an area without the requirement for electronic controls [70]. Passive mechanical elements that operate instantaneously can be employed to distribute mechanical actions in a mechanical interaction, similar to the utilization of cam systems in sports training [71]. Alternatively, yielding elements can be utilized to interrupt the flow of the mechanical energy during the interaction process [72,73].

## 6. Conclusions

This paper proposes a method for properly and generally describing complex interactions among humans, robots, the environment, and objects in collaborative robotic tasks. The proposed method is based on the generalization and proper representation of multiple cooperating reference frame agents at the same time. This allows us to calculate safety indices that provide fast control feedback for adjusting the robot operation in real time. The proposed method is demonstrated with a specific case study that was implemented at the University of Brescia by using a seven-DOF anthropomorphic arm in combination with a specially built psychometric test with multiple human agents. The obtained experimental results are discussed and analyzed to demonstrate the feasibility and effectiveness of the proposed approach for successfully achieving a safe human–cobot interaction.

## Figures and Tables

**Table 1:** Comparison of selected reference frame identifications, selections, and usages.

RF ^1^	Application	MP	AA ^2^	D ^3^	KC	Ref.
A	Autonomous flight	Decouple the trajectory optimization	S	G, T	Dynamically feasible, time-optimal trajectories in the presence of wind.	[22,23,24,25,26,27]
A	Autonomous high-performance flight	Decouple the trajectory optimization	S	G, T	Decouples a path optimization in the ground frame and velocity optimization in the airframe	[28,29,30,31,32]
E,A	Autonomous Mobile Robot	Human/robot distributed control	S	G,T	Definition of perceptive action reference is directly relevant to the measured sensory outputs	[33]
E,A	Autonomous Mobile Robot	Human/robot distributed control	S	G,T	Comparison between perceptive frame and time-based reference frame	[34]
E,A	Spatial mapping	Control of non-holonomic robots	S	G,T	The distance-based holonomic control is transformed to cope with non-holonomic constraints using a piecewise-smooth function	[35,36]
A	Surgical Medical	Graphical User Interface	I	L,T	Development of the frame of reference transformation tool	[37]
E	Human cognition	Human’s perception of spatial relations	S	G, S	Navigation strategies depend on the agent’s confidence (reference frame and sensor information)	[21]

^1^ The reference frame (RF) can be allocentric (A), egocentric (E), or route-centric (R). ^2^ The adaptation ability (AA) can be associated with a sensor in the loop (S), image guidance (I), prior knowledge of the environment (K), or not being available (−). ^3^ The disturbance (D) can be global (G), local (L), time-variant (T), statical (S), or not available (−).

**Table 2:** Classification of interaction cases.

	In	Work	Out	Interactions
(a)	X			1
(b)		X		1
(c)			X	1
(d)	X	X		2
(e)		X	X	2
(f)	X		X	2
(g)	X	X	X	3

**Table 3:** The psychometric scale to measure trust in human–robot collaborations.

Scale Item	Major Components
The way the robot moved made me uncomfortable	Robot’s motion and pick-up speed (Figure 11)
The speed at which the gripper picked up and released the components made me uneasy	
I trusted that the robot was safe to cooperate with	Safe cooperation (Figure 12)
I was comfortable that the robot would not hurt me	
The size of the robot did not intimidate me	
I felt safe interacting with the robot	
I knew the gripper would not drop the components	Robot and gripper reliability (Figure 13)
The robot gripper did not look reliable	
The gripper seemed like it could be trusted	
I felt I could rely on the robot to do what it was supposed to do	

## Data Availability

Data are available under request.

## Nomenclature

**a**	reference frame on a generic object that must be avoided, i.e., anti-task (Section 2);
**a_h_**	reference frame on a generic object that must be avoided, i.e., anti-task, by a generic human agent (Section 2);
**a_r_**	reference frame on a generic object that must be avoided, i.e., anti-task, by a generic robotic agent (Section 2);
**a^i^_j_**	reference frame on the ith object that must be avoided, i.e., anti-task, by a generic jth agent, where i is a natural number and j is a tag that can assume the value h or r, respectively, for a human or robotic agent (Section 2);
**a^i^_j,k_**	reference frame on the ith object that must be avoided, i.e., anti-task, by a generic jth agent, where i is a natural number, j is a tag that can assume the value h or r, respectively, for a human or robotic agent, and kth is a natural number associated with the jth type of agent (Section 2);
**a^i,h^_j_**	reference frame on the ith object that must be avoided, i.e., anti-task, by the hth gripper of a generic jth agent, where i and h are natural numbers and j is a tag that can assume the value h or r, respectively, for a human or robotic agent (Section 2);
**a^i,h^_j,k_**	reference frame on the ith object that must be avoided, i.e., anti-task, by the hth gripper of a generic jth agent, where i and h are natural numbers, j is a tag that can assume the value h or r, respectively, for a human or robotic agent, and kth is a natural number associated with the jth type of agent (Section 2);
a^i^_r(s)_	ith anti-target reference frame perceived by a robot in the topological space (Section 3.1);
a^i^_h(s)_	ith anti-target reference frame perceived by a human in the topological space (Section 3.1);
a_h_	acceleration of the human hand entering the interaction area (Section 3.2);
α	constant parameter incorporating inertia and stiffness of interacting objects (Section 3.3);
**b**	base/absolute reference frame solid with a fixed point in the environment (Section 2);
**b_h_**	base/absolute reference frame solid with a fixed point in the environment for a generic human agent (Section 2);
**b_r_**	base/absolute reference frame solid with a fixed point in the environment for a generic robotic agent (Section 2);
C	constant parameter incorporating uncertainty of the human–robot relative position (Section 4.2);
**d**_i,g_	vector of the minimal distance between the anti-target i and the gripper **g_r_** (Section 3.1);
d_i,g_	module of the vector **d**_i,g_ (Section 3.1);
D_x_	dimension of the interaction area along the *x* axis (Section 3.2);
d_j,k_	distance between the spheres circumscribed to the jth and kth objects (Section 3.3);
**e**	environment; it is used to describe the whole set of objects fixed to the base in the workspace (i.e., the workbench) (Section 2);
ε	measure of the error matrix ∑ (Section 3.1);
ε _xi_	x component of the measurement error of the perceived coordinates x by the ith sensor (Section 3.2);
**g**	reference frame on the hand/gripper of a generic agent (Section 2);
**g_h_**	reference frame on the hand/gripper of a generic human agent (Section 2);
**g_r_**	reference frame on the hand/gripper of a generic robotic agent (Section 2);
**g^i^_j_**	reference frame on the ith hand/gripper of a generic jth agent, where i is a natural number and j is a tag that can assume the value h or r, respectively, for a human or robotic agent (Section 2);
**g^i^_j,k_**	reference frame on the ith hand/gripper of a generic jth agent, where i is a natural number, j is a tag that can assume the value h or r, respectively, for a human or robotic agent, and kth is a natural number associated with the jth type of agent (Section 2);
g_r(s)_	gripper reference frame perceived by a robot in the topological space (Section 3.1);
g_h(s)_	gripper reference frame perceived by a human in the topological space (Section 3.1);
K_H_	human kinematical space in the topological transformation network (Section 3.1);
K_R_	robot kinematical space in the topological transformation network (Section 3.1);
k_h_	human head stiffness (Section 3.3);
k_r_	robot stiffness (Section 3.3);
k	combined stiffness of human and robot (Section 3.3);
M^d^_t,g_	desired (or nominal) orientation matrix between gripper and target object to be manipulated (Section 3.1);
m_h_	human head inertia (Section 3.3);
m_r_	robot inertia (Section 3.3);
**M**_i,j_	transformation matrix from the reference frame (j) to the reference frame (i) (Section 3.1);
**P**_i_	set of homogeneous coordinates describing the position P with respect to the reference frame (i) (Section 3.1);
**P** ^i^_r_	set of homogeneous coordinates describing the position P of the anti-target ith of the robot agent r with respect to the reference frame **a^i^_j_** (Section 3.1);
**P**_g_	set of homogeneous coordinates describing the position P of the anti-target ith of the robot agent r with respect to the reference frame **g_r_** (Section 3.1, Equation (8)); set of homogeneous coordinates describing the position P of the target ith of the robot agent r with respect to the reference frame **g_r_** (Section 3.1, Equation (10));
**P**_t_	set of homogeneous coordinates describing the position P of the target ith of the robot agent r with respect to the reference frame **a_t_** (Section 3.1);
p	payload (Section 3.3);
R_i_	radius of the sphere circumscribing the volume V_i_ (Section 3.1);
R_h_	radius of the sphere circumscribing the human hand (Section 3.2);
**s**	reference frame on a generic sensor of a generic agent (Section 2);
**s_h_**	reference frame on a generic sensor of a generic human agent (Section 2);
**s_r_**	reference frame on a generic sensor of a generic robotic agent (Section 2);
**s^i^_j_**	reference frame on the ith sensor of a generic jth agent, where i is a natural number and j is a tag that can assume the value h or r, respectively, for a human or robotic agent (Section 2);
**s^i^_j,k_**	reference frame on the ith sensor of a generic jth agent, where i is a natural number, j is a tag that can assume the value h or r, respectively, for a human or robotic agent, and kth is a natural number associated with the jth type of agent (Section 2);
S_H_	human sensor, i.e., perceived, space in the topological transformation network (Section 3.1);
S_R_	robot sensor, i.e., perceived, space in the topological transformation network (Section 3.1);
s_r_	reference frame of the perception for a robot sensor in the topological space (Section 3.1);
s_h_	reference frame of the perception for a human sensor in the topological space (Section 3.1);
S_p_	minimum separation distance between human and robot according to ISO/TS 15066 (Section 4.2);
S(t_0_)	human–robot distance at the t0 time;
∑	error matrix describing the difference between the desired and real orientation matrices between the gripper and the target object to be manipulated (Section 3.1);
**t**	reference frame on a generic target (Section 2);
**t_h_**	reference frame on a generic human target (Section 2);
**t_r_**	reference frame on a generic robotic target (Section 2);
**t^i^_j_**	reference frame on the ith target associated with the ith gripper of a generic jth agent, where i is a natural number and j is a tag that can assume the value h or r, respectively, for a human or robotic agent (Section 2);
**t^i^_j,k_**	reference frame on the ith target associated with the ith gripper of a generic jth agent, where i is a natural number, j is a tag that can assume the value h or r, respectively, for a human or robotic agent, and kth is the a natural number associated with the jth type of agent (Section 2);
**T**_i,g_	translational term of the transformation matrix **M**_i,g_ (Section 3.1);
T_j i,g_	jth scalar component along the jth axis of the translational term **T**_i,g_ (Section 3.1);
t_r(s)_	target reference frame perceived by a robot in the topological space (Section 3.1);
t_h(s)_	target reference frame perceived by a human in the topological space (Section 3.1);
T_r_	reaction time of the robot (Section 4.2);
**V**	volume of points solid with the anti-task reference frame that should be avoided (Section 2);
**V^−^**	volume of points solid with the target reference frame that must be considered allow a proper manipulation and to avoid undesired contacts with the target (Section 2);
**V**^j^_i_(**x^j^_i_**)	volume of points solid with the anti-task reference frame of the jth object **x** of the ith agent that should be avoided, where i and j are natural numbers and x can be any object in the workspace that should be avoided (an anti-task, a different gripper, a task of another gripper) (Section 2);
**V**^−,j^_i_(t**^j^_i_**)	volume of points solid with the jth target **t** reference frame of the ith agent that must be considered allow a proper manipulation and to avoid undesired contacts with the target (Section 2);
v_h_	speed of the human hand entering the interaction area (Section 3.2);
v	relative speed between robot and human (Section 3.3);
v_h_	component of the human speed in the direction of the robot (Section 4.2);
sv_r_	component of the robot speed in the direction of the human (Section 4.2).
